# Development and Synthesis of New Therapeutics for Alzheimer’s Disease

**DOI:** 10.1093/geroni/igaf122.3177

**Published:** 2025-12-31

**Authors:** Lilianna Tchatlian, Stevan Pecic

**Affiliations:** California State University, Fullerton, Glendale, California, United States; California State University, Fullerton, Fullerton, California, United States

## Abstract

Alzheimer’s disease (AD) is the sixth leading cause of death among older adults, with 1 in 3 seniors dying with AD or another form of dementia. In 2022, the U.S. had an age-adjusted mortality rate of 28.9 deaths per 100,000 people due to AD. A key therapeutic target for managing AD symptoms is acetylcholinesterase (AChE), an enzyme that breaks down acetylcholine. Donepezil, the most prescribed FDA-approved drug, inhibits AChE but only addresses mild to moderate symptoms in the early stages of AD and is inefficient in stopping the progression of the disease. The long-term goal of our lab is to develop new AChE inhibitors as potential anti-AD therapeutics. In this study we evaluated 25 new compounds, organized in 4 different libraries for their activity in AChE inhibition assay. As part of our drug design process, we first performed several pharmacokinetic predictions. Next, we experimentally determined the solubility, followed by AChE inhibition assay. Finally, using molecular modeling and docking studies, we evaluated the binding modes of the most active analogs. Our structure-activity relationship studies showed that coumarin is better tolerated in the AChE binding site than chromene, yielding low micromolar inhibitors. Modifications at position 3 on the chromene moiety did not significantly affect activity. Pharmacokinetic predictions suggest all new analogs can cross the blood-brain barrier, have moderate oral bioavailability and stability, and are non-toxic at therapeutic doses. The data obtained suggests that these new AChE inhibitors are good drug candidates to be further explored in preclinical studies as AD therapeutics.

